# “Double Bind” with a twist: A corpus-assisted discourse study of gender performances of male and female entrepreneurs on Twitter (now X)

**DOI:** 10.1371/journal.pone.0331400

**Published:** 2025-08-29

**Authors:** Ming Liu, Guofeng Wang, Ruinan Zhao

**Affiliations:** 1 Department of Chinese and Bilingual Studies, The Hong Kong Polytechnic University, Kowloon, Hong KongPeople’s Republic of China; 2 Foreign Languages College, Shanghai Normal University, Shanghai, China; 3 School of Foreign Languages, Guangzhou Maritime University, Huangpu, Guangzhou, China; Universidade Federal do Tocantins, BRAZIL

## Abstract

Previous studies have demonstrated that women in male-dominated fields face a challenging “double bind”—they must display traditionally masculine traits while maintaining feminine qualities. This study provides a corpus-assisted discourse analysis of gender performances by male and female entrepreneurs on Twitter (now X) at three levels: (1) issues, (2) personality traits, and (3) linguistic styles. The primary purpose is to examine how the “double bind” manifests in male and female entrepreneurs’ social media performances. The findings suggest that both male and female entrepreneurs strategically navigate the “double bind”, balancing gender role expectations while engaging their followers across diverse topics. Their performances feature a “double bind” with a twist, as both male and female entrepreneurs tend to adopt an interactive and engaging style rather than a combative and confrontational one to connect with their followers and maintain interactive intimacy.

## 1. Introduction

Previous studies have demonstrated that women in male-dominated fields face a challenging “double bind”—they must display traditionally masculine traits while maintaining feminine qualities [1]. In leadership roles, women who exhibit “toughness” may be criticized for lacking warmth, while those perceived as too feminine may be deemed incompetent to lead [2]. This dynamic is particularly evident in politics, where voters associate different competencies with gender. Male politicians are typically viewed as more capable in areas like economics, military affairs, and technology, while women are often relegated to “softer” domains such as education, healthcare, and social welfare [3]. Female politicians frequently adopt traditionally masculine characteristics to counter these stereotypes [4], while male politicians often emphasize their own softer qualities [5].

In entrepreneurship, success has traditionally been associated with masculine traits like risk-taking, assertiveness, and competitiveness [6]. This association can disadvantage women entrepreneurs, who may be expected to simultaneously demonstrate these qualities while maintaining traditionally feminine characteristics like warmth and relationality [7]. Studies have shown different expectations for male and female CEOs, with women facing additional pressure to demonstrate empathy and human resource management skills [8]. The disparity is particularly evident in how female CEOs are expected to be more caring and empathetic towards employee needs, while male CEOs are primarily evaluated on their business decisions and communication effectiveness. These gendered expectations create additional challenges for women in leadership positions, who must carefully navigate both professional competence and societal expectations of femininity [9].

The rise of social media has introduced new dimensions to this discourse [10]. While some researchers suggest that social media platforms could serve as equalizers for women entrepreneurs by providing new opportunities and reducing traditional barriers [11], others argue that these digital spaces may actually reinforce or amplify existing gender disparities [9,11,12]. The debate continues regarding whether social media truly empowers women in entrepreneurship or merely replicates offline gender biases in digital form [12]. Some studies highlight social media’s potential as a “safe space” where women can overcome traditional obstacles and access new economic opportunities [11,13], while others warn against overemphasizing the empowering effects of social media [9,12]. This complex relationship between gender, entrepreneurship, and social media platforms warrants further investigation. This study integrates corpus linguistics and critical discourse analysis to explore how the “double bind” emerges in the performances of male and female entrepreneurs on social media. The basic assumption is that gender performances of male and female entrepreneurs on social media feature the “double bind” with a twist: while both groups strategically adapt to traditional gender norms to foreground their credibility and competence, the interactive nature of social media also allows them to navigate the “double bind” strategically.

## 2. Cyberfeminism and gender performances on social media

This study is informed by cyberfeminism, which examines the Internet’s impact on gendered power dynamics through the lens of social construction [12,14]. It adopts the perspective that gender is a “social construct” rather than an inherent social category, emphasizing that gender is an active enactment—a matter of “doing” rather than a static state of “being” [15]. Accordingly, gender is perceived as both an outcome and an ongoing process shaped through various forms of expression and self-expression [16]. Within this socially constructed framework, gender identity is a pivotal concept that reflects the degree to which individuals embody masculine and/or feminine characteristics as delineated by conventional gender norms [13]. Thus, the act of constructing gender is inherently tied to power dynamics, a connection that is notably evident in the politics of gender portrayal within social media contexts [17].

The exploration of how women express their gender identities in the digital realm has been a recurring theme in cyberfeminist scholarship [12], reflecting the multifaceted ways in which women navigate the Internet, sometimes challenging but often perpetuating entrenched gender and racial hierarchies [14]. As understanding of the interplay between gender and digital culture evolves, a growing body of cyberfeminist work suggests that socio-economic disparities are often mirrored or even intensified in the online world [18]. The crux of the cyberfeminist debate centers on whether and how Internet technologies can disrupt prevailing gender disparities; this includes an examination of the degree to which inequalities evident in the offline world persist in the digital space [14].

As McAdam et al. [11] review, contemporary research in cyberfeminism pursues two main lines of inquiry. The first echoes the perspective of Plant [19], which posits that the Internet can alter traditional gender power dynamics. Researchers in this vein view the Internet as a “safe space” that provides women the comfort to thrive [11], bolstering women’s self-assurance, enhancing their communication abilities, fostering community engagement, and possibly unlocking economic possibilities. The second line of critique, however, questions the neutrality of the Internet as an entrepreneurial platform [20], and warns that the capacity of digital technology to empower economically marginalized women might be exaggerated, given that gender inequalities encountered offline tend to be replicated in the online environment [12].

While traditional studies used to conceptualize masculinity and femininity as bipolar ends of a single continuum and view women’s and men’s expected ways of thinking and behaving as either masculine or feminine [21], cyberfeminist research has underlined that a person can simultaneously possess and exhibit femininity and masculinity [14]. For example, a person scoring high in one dimension (i.e., femininity or masculinity) would exhibit the stereotypes of that gender (masculine or feminine) identity. The cyberfeminist theory expects that women engaged in digital space could exhibit traits that are both masculine (e.g., aggressive, assertive, and forceful) and feminine (e.g., affectionate, warm, and yielding), both instrumental (e.g., getting the thing accomplished) and expressive (e.g., sensitive to the needs of others) [22]. However, there remains a scarcity of research that analyzes the linguistic expressions of gender by both male and female entrepreneurs on social media.

## 3. From critical discourse analysis to corpus-assisted discourse studies

Critical discourse analysis (CDA) views discourse as a social practice and underlines the importance of examining discourse in its socio-political contexts [23]. It aims to expose the relations between language, power, and society [24]. CDA views gender as socially constructed and aims to explicate the “(re)production, negotiation, and contestation of gender ideologies and power relations in a particular context” [25]. It has been employed to uncover the construction of gender identities and dissemination of gender norms and roles [26]. However, CDA used to rely on a qualitative of small samples of texts, which has been criticized for a lack of methodological rigor and interpretation problems [27].

The recent development of corpus linguistics (CL) has contributed to growing studies in combining CL and CDA, but most of these studies fail to give a balanced combination of both disciplines [28]. Corpus-assisted discourse studies (CADS) is known for its emphasis on the balanced ‘synergy’ of quantitative corpus linguistic methods with detailed qualitative discourse analysis [29]. On the one hand, CL can benefit CDA by processing large samples of data efficiently and identifying language patterns that cannot be obtained through mere manual analysis. On the other hand, CDA can contribute to better interpretation and explanation of the findings generated by corpus analytic tools. CADS has been widely applied to the analysis of discursive contractions of different topics including war and politics [30–32], immigrants and minority groups [28], environment [33,34], social movement and protests [35].

It has also been used in the analysis of discursive constructions of gender [36]. However, these studies used to rely on traditional CL methods, such as keywords, collocates, and concordance [37], and more efforts are needed to take advantage of recent development in computational linguistics and text mining to realize the accurate processing of large samples of texts [38]. Newman et al. [38] compared the language of men and women across 14,000 samples of Twitter texts with the Linguistic Inquiry and Word Count tool. Windsor et al. [39] use a suite of computational linguistic approaches such as topic modelling, sentiment analysis and model the evolution of gendered language in the US senate. These studies have demonstrated the analytic potentials of quantitative analytic methods in revealing the linguistic differences of different genders.

## 4. Data collection and analytic methods

### 4.1. Data collection

Twitter, now known as X, stands as one of the world’s preeminent microblogging platforms, with around 600 million monthly users as of April 2025 [40]. The platform serves as a crucial channel for social connection and interaction, enabling users to communicate through concise messages of up to 280 characters [4]. Recent research indicates that social media platforms have emerged as supportive environments for female users, facilitating self-promotion and follower engagement strategies [41].

This study analyzes the 100 most recent posts from each of top 10 influential entrepreneurial male and female leaders, as ranked by Forbes in its “The Most Powerful Women List” (2021) and “The World’s Billionaire List” (2021). The inclusion of top 10 influential entrepreneurial leaders ensures that the sample is comprised of verified, credible figures in business and entrepreneurial contexts. Furthermore, the more influential these female entrepreneurs become, the more likely they are to experience the “double bind,” making their discourse particularly relevant for analysis.

Table 1 provides a detailed overview of their Twitter accounts, showcasing the names, positions, and number of followers at the time of data collection, which further validates the sample’s credibility. Data collection was conducted using the NCapture function within NVivo software, a tool specifically designed to streamline the process of gathering data from various sources. NVivo’s NCapture function utilizes Twitter’s API (Application Programming Interface) to access and collect data efficiently.

**Table 1:** Overview of entrepreneurs’ Twitter accounts (elaborated by the authors).

Gender	Rank	Name	Country	Position	Followers
F	1	Mary Barra	United States	CEO of General Motors	63.2k
	2	Melinda French Gates	United States	Cochair of Bill & Melinda Gates Foundation	2.5m
	3	Julie Sweet	United States	Chair & CEO of Accenture	31.1k
	4	Susan Wojcicki	United States	CEO of YouTube	358.8k
	5	Oprah Winfrey	United States	Entrepreneur, Philanthropist	43.2m
	6	Cathie Wood	United States	Founder of Ark Invest	1.4m
	7	Kiran Mazumdar-Shaw	India	Chair of Biocon	1.6m
	8	Reese Witherspoon	United States	Film Producer	2.9m
	9	Kirsten Green	United States	Founder of Forerunner Ventures	37.4k
	10	Adena Friedman	United States	President of Nasdaq	17.8k
M	1	Tim Cook	Unites States	CEO of Apple	13.5m
	2	Elon Musk	Unites States	founder, CEO, and Chief Engineer at SpaceX	102m
	3	Jeff Bezos	Unites States	founder, CEO of Amazon	4.9m
	4	Bill Gates	Unites States	Co-founder of Mircrosoft	60m
	5	Mike Bloomberg	Unites States	co-founder and CEO of Bloomberg L.P.	2.7m
	6	Changpeng Zhao	Chinese-Canadian	CEO of Binance	6.6m
	7	Michael Dell	United States	CEO of Dell	676.5k
	8	Ray Dalio	Unites States	founder of Bridgewater Associates	1m
	9	Pavel Durov	Russian-born Emirati	founder of the social networking site VK and Telegram Messenger	1.3m
	10	Uday Kotak	India	CEO of Kotak Mahindra Bank	1.1m

The Twitter (X) usernames of the top 10 male and female entrepreneurs were used as input search parameters, with the data collection configured to include only the 100 most recent posts from each account. To minimize data redundancy and ensure a diverse representation of daily topics, only the initial post of each day was selected [42]. This methodological decision allows for a focus on the entrepreneurs’ most deliberate and unprompted communications of the day, avoiding potential influence from interactions or reactions later in the day.

The resulting corpora consist of 31,124 words from female entrepreneurs and 27,773 words from male entrepreneurs, providing a robust dataset for analyzing gendered discourse patterns in entrepreneurial contexts. The collection and analysis method complied with the terms and conditions for the source of the data.

### 4.2. Analytical framework and procedures

This study aims to analyze gender performances on Twitter at multi-levels of discourse: (1) (masculine vs. feminine) issues; (2) (masculine vs. feminine) personality traits; (3) masculine vs feminine linguistic style [38,41,43]. So-called masculine issues that are stereotypically assumed to be better handled by men include economy, military, foreign affairs, technology, science, crime, terrorism, and gun problems, while so-called feminine issues for which women are perceived to be more suitable include education, health, human right, women’s rights, animal right, child care, poverty, arts, environment, and social welfare [41,44]. Traditional social role theory posits that men and women are assumed to have different personality traits; men are expected to be strong, tough, assertive, competent, instrumental, and achievement-oriented (so-called masculine traits), whereas women are assumed to be warm, caring, understanding, compassionate, expressive, and family-oriented [41].

Contemporary computational literature often distinguishes men and women on pragmatic dimensions of “informativeness” and “involvement” based on earlier corpus-based contrasts of written and spoken genres [45]. The involvement dimension consists of linguistic resources that create interactions between speakers and their audiences; the informational dimension consists of resources that communicate propositional content. Early work compared frequencies of large word classes, such as parts-of-speech: the involvement dimension includes first and second person pronouns, present tense verbs, and contractions [45,46], while the informational dimension includes prepositions, attributive adjectives, and longer words. Informational word classes were used preferentially by men, while involvement and interaction are associated with women [47].

The online corpus-analytic tools Wmatrix 5.0 and KH Coder were used in this study to examine whether and to what extent male and female entrepreneurs vary in the choice of issues, personality traits and linguistic styles. Wmatrix 5.0 is a software for corpus analysis and comparison. It provides a web interface to natural language processing tools such as the USAS and CLAWS corpus annotation tools for English. USAS (UCREL Semantic Analysis System) is a framework for automatic semantic analysis of texts. It has a multi-tier structure with 21 major semantic fields and 232 sub-semantic categories. CLAWS (the Constituent Likelihood Automatic Word-tagging System) is a Part-of-Speech (POS) tagging tool for English texts.

With its corpus annotation tools, Wmatrix can extend keyword analysis to the analysis of key semantic categories (SMCs) and key parts of speech (POSs) [48]. Keyness is measured by log-likelihood (LL) values: the higher the keyness values, the more statistically significant the differences [48]. The two corpora were first compared with a general reference corpus, American English 2006 (AmE06), to generate two key SMC lists. The top 20 key SMCs were compared to identify those present in both corpora. Then, the two corpora were compared with each other to generate two additional key SMC lists which can suggest their preferential key SMCs. This study focuses on the top 20 preferential key SMCs of each corpus and identify their respective preferences for topics and personality traits. In order to guarantee that those key SMCs identified are statistically significant, the LL values of these key SMCs must be above 6.63 (p < 0.01). Additionally, the two corpora were compared to generate two key POS lists. The key POSs with LL values above 6.63 (p < 0.01) were analyzed to identify the distinctive POSs of the two corpora [49]. These findings suggest the characteristic linguistic styles of each corpus.

This study employs KH Coder, an open-source text mining software, to analyze variations in self-presentation between female and male entrepreneurs. Originally developed for quantitative content analysis, KH Coder integrates the Stanford POS Tagger for English word extraction, R for statistical computations, and MySQL for data management and retrieval [50]. The software offers a range of analytic tools, including frequency analysis, concordance examination, key term extraction, word association mapping, correspondence analysis, multi-dimensional scaling, hierarchical clustering, co-occurrence network visualization, and topic modeling [50,51].

For this analysis, we conducted word association analysis to examine terms frequently co-occurring with first-person pronouns (*I*, *we*, *our*) within a sentence. KH Coder visualizes these co-occurrence patterns through network diagrams, with line thickness indicating the strength of word associations. By default, the software displays the 60 strongest word pair connections, omitting unconnected terms from the visualization [50]. Within these networks, the software automatically identifies and color-codes distinct word clusters, revealing thematic patterns in how entrepreneurs represent themselves in their communications.

## 5. Data analysis and discussion

### 5.1. Analysis of shared key semantic categories

An analysis of the top 20 key semantic categories (SMCs) of both corpora (see Table 2) when they are compared with American English 2006 (AmE06) reveals 12 shared key SMCs. These include “Helping” (S8+), “Unmatched” (Z99), “Content” (E4.2+), “The universe” (W1), “Polite” (S1.2.4+), “Time: New and young” (T3-), “Time: Future” (T1.1.3), “Belonging to a group” (S5+), “Evaluation: Good” (A5.1+), “Money and pay” (I1.1), and “Time: Present; simultaneous” (T1.1.2). The I1.1 category, comprising terms such as *stakeholders*, *investors*, *invest(ing)*, *investment*, and *capital*, reflects the expected financial focus of entrepreneurial discourse.

**Table 2:** Top 20 Key semantic categories (Elaborated by the authors).

	Female entrepreneurs			Male entrepreneurs		
Rank	Tagset	LL	Semantic category	Tagset	LL	Semantic category
1	S8+	242.49	Helping	W1	203.32	The universe
2	Z99	167.15	Unmatched	S1.2.4+.	163.18	Polite
3	E4.2+	165.22	Content	S8+	133.61	Helping
4	I2.1	132.77	Business: Generally	T1.1.3	124.72	Time: Future
5	W1	126.52	The universe	Q1.3	117.14	Telecommunications
6	S1.2.4+.	125.5	Polite	A5.1+	109.78	Evaluation: Good
7	T3-	113.49	Time: New and young	E4.1+	99.92	Happy
8	T1.1.3	112.54	Time: Future	I1	88.69	Money generally
9	E2+	92.72	Like	X9.2+	80.79	Success
10	S5+	92.26	Belonging to a group	E4.2+	78.57	Content
11	A5.1+	89.29	Evaluation: Good	Z99	78.06	Unmatched
12	I3.1	88.05	Work and employment: Generally	I1.3+	61.97	Expensive
13	I1.1	81.76	Money and pay	I1.1	59.12	Money and pay
14	T1.1.2	77.94	Time: Present; simultaneous	T3-	55.07	Time: New and young
15	S1.1.2+.	73.95	Reciprocal	S3.1.	43.59	Personal relationship: General
16	I2.2	71.05	Business: Selling	T1.1.2	43	Time: Present; simultaneous
17	A11.1+	66.79	Important	W4	42.37	Weather
18	Y1	56.09	Science and technology in general	S5+	39.97	Belonging to a group
19	E4.1+	55.23	Happy	A5.1+++	39.19	Evaluation: Good
20	A1.4	49.86	Chance, luck	S8-	38.07	Hindering

Both gender groups exhibit similar behavioral patterns in their communications, demonstrating caring (S8+), nurturing (A5.1+ and S1.1.2+), and emotional expressiveness (E4.2+ and E4.1+) [52]. The helping orientation (S8+) is particularly prominent across both groups. In the female entrepreneurs’ corpus, this manifests through terms like *help* (60), *support* (30), *helping* (25), *inspiring* (19), *services* (11), and *supporting* (11). The male entrepreneurs’ corpus shows similar patterns with *help* (46), *support* (31), *helping* (11), *inspiring* (10), *protecting* (10), and *helped* (10). These linguistic choices position both groups as helpers and supporters within their professional spheres.

Both male and female entrepreneurs express strong positive emotions in their communications, particularly through expressions of happiness (**E4.1+**) and satisfaction (**E4.2+**). The satisfaction category is dominated by words like *proud*, *glad*, and *pleasure*, while happiness is communicated through terms like *happy*, *celebrating*, and *celebrate*. This can be attributed to the curtesy on social media to celebrate and share good news. They also share a tendency toward positive evaluation (**A5.1+**), frequently using words such as *great*, *progress*, *well*, and *good*. These positive evaluative words suggest that both groups feel confident and satisfied with their achievements.

The analysis reveals that both female and male entrepreneurs foreground politeness (**S1.2.4+**). Female entrepreneurs use terms like *thanks* (32 occurrences) and *grateful* (14), while male entrepreneurs show comparable usage with *thanks* (41) and *grateful* (17). This suggests a shared recognition of the importance of maintaining professional courtesy and expressing appreciation.

Time-related themes emerge as another significant commonality across both groups, which can be seen from three distinct temporal categories. References to newness and youth (**T3-**) appear frequently, with *new*, *young*, and *innovation* being prominent terms. Future-oriented language (**T1.1.3**) is evident through frequent use of *will*, *future*, and *tomorrow*, while present-focused communication (**T1.1.2**) employs terms like *today* and *now*. This temporal focus suggests both groups are actively engaged in innovation and forward-thinking initiatives while maintaining awareness of current developments.

Despite these shared patterns, they also show some gender-specific preferences for some key SMCs. Female entrepreneurs demonstrate a stronger focus on business-related topics (**I2.1**), work and employment (**I3.1**), sales (**I2.2**), and science/technology (**Y1**). Male entrepreneurs, conversely, show greater emphasis on telecommunications (**Q1.3**), general money matters (**I1**), and weather-related topics (**W4**). However, in order to examine whether they show statistically significant differences in the choice of these SMCs, the following section compares the two corpora with each other to identify their preferential key SMCs.

### 5.2. Analysis of preferential key SMCs

Table 3 shows the top 20 preferential key SMCs when comparing male and female entrepreneurs. The analysis of topic preferences reveals distinct patterns between female and male entrepreneurs. Female entrepreneurs show preferences for traditionally feminine topics, as evidenced by several key semantic categories. Their discourse prominently features references to women and gender (**S2.1**) with terms like *women* (67 occurrences) and *female* (5), musical themes (**K2**) including *music* (7) and *musical* (6), family relationships (**S4**) such as *family* (37) and *mother* (4), and work-related topics (**I3.1**) with terms like *work* (76) and *career* (9). This pattern aligns with Hu and Kearney’s [53] observation that despite increasing female participation in the workforce, traditional social role divisions persist, with women maintaining stronger connections to family and domestic spheres.

**Table 3:** Top 20 preferential key SMCs (Elaborated by the authors).

Rank	Female entrepreneurs			Male entrepreneurs		
	Tagset	LL	Semantic category	Tagset	LL	Semantic category
1	S2.1.	71.03	People: Female	Q1.3	73.59	Telecommunications
2	Y1	32.55	Science and technology in general	Z2	51.88	Geographical names
3	Z5	20.51	Grammatical bin	I1	49.98	Money generally
4	M3	17.76	Vehicles and transport on land	Z6	40.42	Negative
5	Z4	15.04	Discourse Bin	A1.5.1	36.19	Using
6	S1.1.2+.	14.45	Reciprocal	G2.2	30.48	General ethics
7	I2.1	13.67	Business: Generally	P1	26.85	Education in general
8	Q4.1	13.61	The Media: Books	A12+	18.51	Easy
9	Q2.1	13.38	Speech: Communicative	W4	18.43	Weather
10	K2	13.22	Music and related activities	F3	16.76	Smoking and non-medical drugs
11	A6.3+	11.44	Comparing: Varied	A10-	16.68	Closed; Hiding/Hidden
12	A1.8+	10.77	Inclusion	G1.1	15.17	Government
13	X2.4	10.51	Investigate, examine, test, search	T1	15.14	Time
14	S4	9.91	Kin	A3+	13.02	Existing
15	E2+	9.81	Like	O2	12.27	Objects generally
16	S7.1+.	9.78	In power	S7.4+.	12.05	Allowed
17	X3.4	9.59	Sensory: Sight	A6.2+	11.58	Comparing: Usual
18	I3.1	8.21	Work and employment: Generally	I1.2	11.18	Money: Debts
19	T3++	7.55	Time: Old; grown-up	Z7	10.68	If
20	X9.1+	7.3	Able/intelligent	X6	10.67	Deciding

However, female entrepreneurs also demonstrate significant engagement with traditionally masculine topics. This is evident in their focus on science and technology (**Y1**) using terms like *technology* (39) and *engineering* (5), business matters (**I2.1**) with references to *business* (39) and *companies* (32), and leadership (**S7.1+**) through words like *leadership* (28) and *CEO* (13).

Male entrepreneurs, for their part, emphasize distinctly masculine topics in their communication. Their discourse centers on telecommunications (**Q1.3**) with frequent mentions of *telegram* (61), financial matters (**I1**) including *money* (14) and *finance* (7), health-related issues like *smoking* (F3), and governmental affairs (**G1.1**) with references to *government* (10) and *President* (10). They also show particular attention to financial transactions (**I1.2**) through terms like *pay* (6) and *spend* (6).

Interestingly, male entrepreneurs also engage with traditionally feminine topics, particularly in two areas: education (**P1**), using terms like *students* (13) and *education* (10), and weather-related topics (**W4**), with frequent references to *climate* (41) and various weather phenomena. This suggests that while gender-based topic preferences persist, there is some degree of cross-over in both directions.

The analysis reveals a nuanced pattern of topic preferences that transcends traditional gender expectations. Female entrepreneurs demonstrate strong engagement with traditionally masculine domains like technology and business, while maintaining presence in feminine-associated areas like music and employment. Similarly, male entrepreneurs show significant activity in masculine-coded areas like finance while also engaging with traditionally feminine domains such as education and environmental topics. This mixed engagement challenges traditional gender-based competency perceptions where men are typically associated with economy, military, technology, science, and security issues, while women are typically associated with education, healthcare, human rights, social welfare, and environmental concerns [44]. The findings suggest that both male and female entrepreneurs strategically navigate the “double bind”, balancing gender role expectations while engaging their followers across diverse topics. Rather than strictly adhering to gender-typical domains, both male and female entrepreneurs demonstrate flexibility in topic selection, perhaps reflecting the evolving nature of entrepreneurial leadership in contemporary contexts.

The key SMC analysis also reveals distinct communication patterns among female entrepreneurs that align with traditional gender-based leadership expectations [3]. Their language choices reflect traditionally feminine personality traits, particularly in three key areas of interpersonal communication, community orientation, and emotional expression.

In terms of discourse markers (**Z4**), female entrepreneurs show a strong preference for gratitude and supportive expressions, with *thank_you* appearing 90 times, followed by encouraging phrases like *please* (5), *bravo* (5), and *well_done* (4). This emphasis on acknowledgment and appreciation suggests a conscious effort to build and maintain relationships with followers.

The prominence of reciprocity-related terms (**S1.1.2+**) demonstrates a clear community orientation, with frequent use of sharing-related vocabulary (*share*: 33, *sharing*: 15, *shared*: 15). Similarly, their use of positive affect terms (**E2+**) is characterized by strong emotional expression through words like *love* (41), *loved* (17), *enjoyed* (10), and *appreciate* (6).

These patterns align with previous research findings that women tend to reference emotions more frequently than men [54] and show higher use of positive emotional expression [55]. These linguistic choices reinforce the construction of female entrepreneurs as more emotionally expressive, relationship-focused, and community-oriented than their male counterparts [56], particularly evident in their frequent use of gratitude expressions and positive emotional language.

Female entrepreneurs demonstrate a distinctive approach to follower relationships, characterized by an emphasis on collaborative investigation and inclusive observation. This is evident in their language patterns across three key semantic categories: research-oriented terms (**X2.4**) such as *research* (19) and *analysis* (4); diversity-focused language (**A6.3**) including *diversity* (13) and *diverse* (5); and observational verbs (**X3.4**) like *see* (67) and *watch* (13).

This communication style presents a notable contrast to female politicians, who often adopt more masculine, combative approaches in their campaigns [5,57]. Instead, female entrepreneurs favor cooperative and relationship-focused communication strategies with their followers. They tend to show a reluctance to impose their views on others.

Male entrepreneurs exhibit communication patterns that align with traditional masculine personality traits [58]. Their language choices reflect several distinct characteristics: competitiveness and confrontation (evidenced in negative markers like *n’t* [89], *not* [68], *no* [18] in **Z6**), task orientation (shown through terms like *principles* [13] and *objective* [2] in **G2.2**), and security consciousness (reflected in words like *privacy* [11] and *encrypted* [2] in **A10-**).

A notably impersonal communication style emerges through their frequent use of existential verbs (**A3+**) such as *is* (283), *are* (104), and *be* (83). This is further reinforced by their tendency to reference general objects (**O2**), using terms like *things* (25), *products* (11), and *tools* (5). These patterns collectively construct an image of male entrepreneurs as emotionally controlled, assertive, and authoritative [56].

The contrast between male and female entrepreneurial communication styles is striking. While female entrepreneurs tend toward relational and people-oriented approaches [58], male entrepreneurs focus more on objects and impersonal topics [59]. Female entrepreneurs often emphasize work-related topics and consciously incorporate masculine traits while maintaining attention to women’s issues. In contrast, male entrepreneurs engage with a broader spectrum of challenging topics from education and climate to economics and politics, approaching these subjects from a notably critical perspective. Unlike their female counterparts, male entrepreneurs appear less concerned with consciously displaying gender-typical traits, instead maintaining their focus on objective and impersonal communication styles.

### 5.3. Analysis of key part-of-speech (POS) categories

The analysis of key Parts of Speech (POS) categories, evaluated at a statistical significance level of *p* < 0.01 (cutoff value 6.63), reveals distinct linguistic patterns between male and female entrepreneurs (see Table 4). A notable finding is the disparity in distinctive POS categories—female entrepreneurs exhibit only 7 categories compared to male entrepreneurs’ 15. This suggests that female entrepreneurs tend to adopt communication styles that more closely align with male patterns.

**Table 4:** Preferential key POSs of the two corpora (Elaborated by the authors).

Types	Rank	Item	Freq.	LL	Key POS	Tokens
M	1	XX	159	38.18	not, n’t	*n’t* (89), *not* (68), *not_only* (1), *not_a_single* (1)
	2	RR	544	37.57	general adverb	*just* (41), *never* (25), *so* (22), *always* (20), *ever* (19)
	3	VD0	62	24.15	do, base form (finite)	*do* (61), *do_you_think* (1)
	4	CS	154	19.88	subordinating conjunction	*if* (49), *when* (28), *because* (18), *while* (14), *since* (10)
	5	VDI	30	11.86	do, infinitive	*do* (30)
	6	DD2	63	10.74	plural determiner	*these* (40), *those* (23)
	7	MC	252	10.52	cardinal number, neutral for number	*covid-19* (12), *2022* (10), *3* (9), *2021* (9), *10* (8)
	8	NN	150	10.06	common noun, neutral for number	*people* (86), *data* (25), *media* (15), *$* (6), *species* (3)
	9	VDZ	15	8.33	*does*	*does* (15)
	10	NP1	647	7.98	singular proper noun	*India* (47), *US* (28), *China* (13), *Ukraine* (11)
	11	PPHO2	36	7.85	3rd person plural objective personal pronoun	*them* (36)
	12	REX	5	7.62	adverb introducing appositional constructions	*e.g.,* (3), *i.e.,* (1), *i.e.,* (1)
	13	VVZ	257	7.49	s form of lexical verb (e.g., *gives*, *works*)	*shows* (8), *means* (6), *lives* (6), *needs* (6), *makes* (5)
	14	NNU	127	7.44	unit of measurement, neutral for number (e.g., in, cc)	*100%* (6), *$100m* (4), *10%* (3), *1%* (3), *bn* (3), *2yr* (2)
	15	JJ	1982	6.89	general adjective	*new* (71), *great* (60), *good* (32), *pandemic* (32), *proud* (31)
F	1	APPGE	816	53.41	possessive pronoun, pre-nominal	*our* (352), *my* (121), *your* (109), *their* (94), *her* (62), *his* (46)
	2	VVG	736	14.88	ing participle of lexical verb	*helping* (23), *making* (23), *working* (21), *creating* (18)
	3	NN1	4347	14.63	singular common noun	*world* (87), team (84), *future* (56), *work* (49), *life* (40)
	4	II	1834	11.99	general preposition	*in* (417), *to* (401), *on* (239), *at* (137), *about* (107), *by* (93)
	5	IF	441	9.87	for (as preposition)	*for* (438)
	6	AT1	628	7.73	singular article	*a* (469), *an* (109), *every* (28), *every_day*(11), *a_lot* (4)
	7	PPHS1	56	6.86	3rd person sing. subjective personal pronoun	*she* (31), *he* (25)

Female entrepreneurs demonstrate characteristic usage of specific linguistic elements: possessive pronouns (APPGE), third-person singular pronouns (PPHS1), prepositions (II and IF), verb participles (VVG), singular common nouns (NN1), and articles (AT1). This pattern, particularly the prominence of pronouns, aligns with previous research identifying pronouns as typical markers of female authorship [43]. While the use of possessive pronouns might suggest an involved communication style, this characteristic appears relatively modest in the analysis.

Male entrepreneurs, by contrast, demonstrate a more information-focused communication style. Their language is characterized by diverse verb forms (VVZ, VD0, VDI, VDZ), conjunctions (CS), numerical expressions (MC, NNU), general adjectives (JJ), adverbs (RR), and pronouns (NN, NP1). Notably, their use of negation words suggests a tendency toward critical engagement and counterarguments [54].

### 5.4. Word association analysis

**Fig 1:**
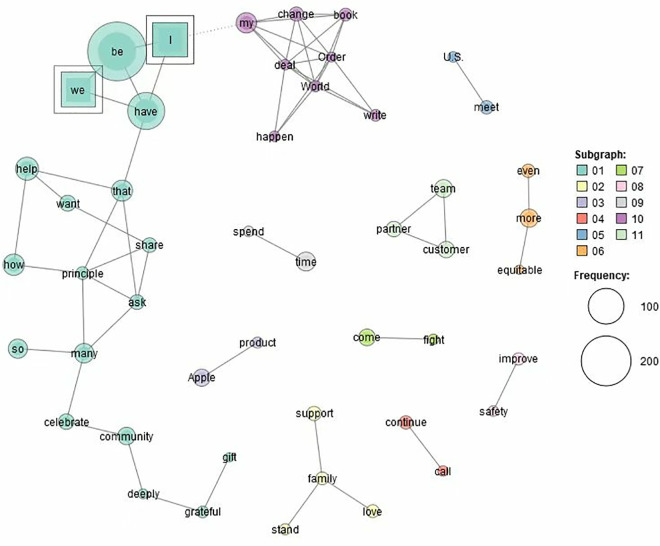
Co-occurring words of first-person pronouns in ME.

**Fig 2:**
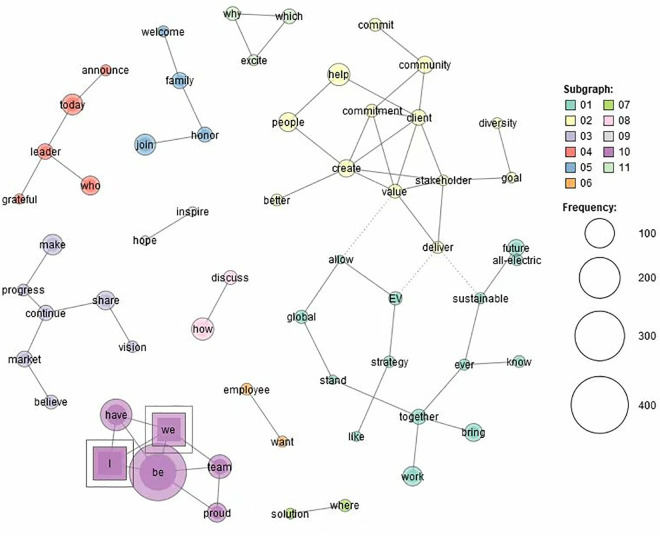
Co-occurring words of first-person pronouns in FE.

Figs 1 and 2 (Generated by the KH Coder) show the words which strongly co-occur with first person pronouns (*I*, *we*, and *our*) in the two corpora. Although both corpora have identified 11 themes, the two corpora show their different preferences for constructing male and female entrepreneurs.

In Fig 1, the most prominent theme is **Theme 1**. It consists of such words as *I*, *we*, *be*, *that*, *have*, *that*, *want*, *help*, *share*, *principle*, *ask*, *so*, *many*, *celebrate*, *community*, *deeply*, *grateful*, and *gift*. It suggests that male entrepreneurs tend to foreground themselves in sharing principles, celebrating achievement and showing gratitude. However, in Fig 2, *I* and *we* only appear in **Theme 10**, which consists of *I*, *we*, *have*, *be*, *team*, and *proud*. They foreground female entrepreneurs’ pride to be a team.

The different themes also suggest their different preferences for topics/issues. Male entrepreneurs tend to foreground the feminine topics of family support (**Theme 2**), equity (**Theme 6**), safety improvement (**Theme 8**), team and partnership (**Theme 11**). Besides, they also foreground the masculine topics of politics (**Theme 10**) and science and technology (**Theme 3**). Male entrepreneurs tend to underline the action taken to achieve the goals, as can be seen from **Theme 4** (*continue* and *call*), **Theme 5** (*meet* and *US*), **Theme 7** (*come* and *fight*), and **Theme 9** (*spend* and *time*).

By contrast, female entrepreneurs show their preferences for the masculine topics of science and technology (**Theme 1**), market (**Theme 3**), and leadership (**Theme 4**). However, they also address some feminine topics, including community development and stakeholder value (**Theme 2**), family (**Theme 5**), and employees (**Theme 6**). Nevertheless, instead of foregrounding action, female entrepreneurs tend to foreground emotion and belief, as can be seen from such words as *want*, *allow*, *commit*, *commitment*, *excite*, *welcome*, *honor*, *proud*, *believe*, *announce*, *discussion*, *inspire* and *hope*.

## 6. Discussion and conclusion

Therefore, the findings reflect a “double bind” with a twist in the gender performances of male and female entrepreneurs on social media, where both groups strategically adapt to traditional gender norms while leveraging social media’s interactive nature. Both male and female entrepreneurs emphasize helping, emotional expressiveness, gratitude, and future-oriented language. These shared performances demonstrate how social media encourages interactive and collaborate communication styles, which partially dissolves traditional gendered expectations of assertiveness for men and emotional expressiveness for women. This adaptive approach reflects the “twist”, where social media allows for blending traditionally masculine and feminine traits. Unlike female politicians who are supposed to adopt a more combative masculine style to foreground their competence and leadership [60], female and male entrepreneurs do not rely on confrontation but interaction and engagement to build connections and solidarity with their followers.

This can be seen from their topic preferences. Female entrepreneurs show strong preferences for traditionally feminine topics like family and community while also engaging with masculine-coded domains such as technology, business, and leadership. This reflects their need to balance feminine and masculine traits to gain credibility while maintaining likability. Male entrepreneurs, conversely, emphasize masculine topics like finance, politics, and science while also engaging with feminine themes such as education, climate, and equity. This suggests that male entrepreneurs tend to adopt traits associated with femininity to enhance follower engagement and relatability.

Their performances in personality traits and styles show even more complicated pictures. Despite both genders’ shared performances, Twitter, as a social media platform, continues to reproduce traditional gender stereotypes about gender-related personality traits [56]. Female entrepreneurs tend to foreground emotions (e.g., pride, commitment, and inspiration) and relational values, reinforcing their alignment with traditional feminine traits. Male entrepreneurs, on the other hand, emphasize actions and achievements, reflecting traditional masculine traits of goal orientation and assertiveness. Despite this, male entrepreneurs also integrate emotional elements like gratitude and community-building, showcasing a nuanced adaptation of feminine communication styles.

Therefore, both male and female entrepreneurs navigate the digital double bind strategically. Female entrepreneurs face the challenge of balancing professional credibility with societal expectations of femininity. They engage in “soft self-promotion” by emphasizing community and emotional values while subtly asserting their leadership and expertise. Male entrepreneurs must also navigate a version of the double bind, as adopting excessively feminine traits could undermine their credibility. Both groups strategically adapt their communication to build professional authority while maintaining relatability.

To sum up, the above findings suggest that while traditional gender norms persist in shaping entrepreneurial communication [56], social media provides a platform for more flexible and nuanced performances. By revealing the “double bind” with a twist, this study highlights how male and female entrepreneurs strategically blend masculine and feminine traits to meet the complex demands of digital entrepreneurship. This suggests a gradual evolution in gendered communication, where traditional norms are negotiated rather than entirely overturned [9].

This analysis ultimately challenges the traditional binary treatment of gender in computational models, suggesting that gender categories are normative rather than merely descriptive [43]. While gender remains a powerful force in structuring social interactions, its expression is more nuanced and complex than simple binary categories suggest, particularly in the digital entrepreneurial space. As Hu and Kearney [53] argue, gender differences shouldn’t be viewed purely negatively, but rather as potential vehicles for women to represent their distinct values and increase their visibility within the entrepreneurial space. This perspective suggests that the goal should not necessarily be complete homogeneity in entrepreneurial discourse, as such homogeneity might simply reflect the forced accommodation of marginalized groups to dominant norms. Instead, the preservation of certain differences might serve as a means for women to maintain their authentic voices while navigating the complex demands of digital entrepreneurship. Therefore, gender performances in other professions are worthy of a close examination in future studies [60].

## Supporting information

S1 File Corpora. (ZIP)
